# Complex forearm deformities: operative strategy in posttraumatic pathology

**DOI:** 10.1007/s11678-015-0341-1

**Published:** 2015-10-14

**Authors:** Konrad Mader, Marianne Koolen, Mark Flipsen, Arnard van der Zwan, Dietmar Pennig, John Ham

**Affiliations:** 1 Orthopædic Department, Section Upper Extremity, Asklepios Hamburg Altona, Paul-Ehrlich-Straße 1, 22763 Hamburg, Germany; 2 Department of Surgical Sciences (K1), University in Bergen, Bergen, Norway; 3 Orthopædic Department, Onze Lieve Vrouwe Gasthuis, Amsterdam, The Netherlands; 4 Klinik für Unfallchirurgie/Orthopädie, Hand- und Wiederherstellungschirurgie, St. Vinzenz- Hospital, Köln, Germany

**Keywords:** Posttraumatic deformity forearm, Corrective osteotomy, External fixation, Computerized planning, Erworbene Fehlstellung Unterarm, Korrekturosteotomie, Fixateur externe, Computerisierte Planung

## Abstract

Complex posttraumatic forearm deformities have a significant impact on the integrity of the upper extremity leading to pain, instability in both the proximal and/or distal radioulnar articulation, and reduced range of forearm motion. Corrective osteotomy or more advanced procedures for malunited fractures or other posttraumatic deformities of the upper extremity, especially in the forearm are challenging procedures. In this review we will discuss the essential aspects of anatomy and pathomechanics, clinical and radiological assessment and the pathway from preoperative planning to the actual deformity correction surgery, either with one-stage correction or using gradual lengthening with external fixation (“callotasis techniques”) and finally the functional outcome we can expect for our patients. In addition we will analyze the modern computer-assisted techniques available to date.

## Abstract (de)

Komplexe posttraumatische Deformitäten im Bereich des Unterarmeshaben einen signifikant negativen Effekt auf die funktionelle Integrität der betroffenen oberen Extremität. Sie können Schmerzen, Instabilität im proximalen/und oder distalen Radioulnargelenk und eine bedeutende Bewegungseinschränkung verursachen. Korrektureingriffe in diesem Indikationsberich sind gerade im Bereich des Unterarms aufgrund seiner komplexen Pathoanatomie eine echte Herausforderung für den behandelnden Chirurgen. In diesem Übersichtsartikel versuchen wir die essentiellen Aspekte der anatomischen Besonderheiten, der Pathomechanik, die klinischen und radiologischen Untersuchungen und schlieβlich den Weg von der präoperativen Planung über den geeigneten Korrektureingriff (als einzeitige Korrektur mit oder ohne aufwendige computergestützte Planung oder graduell mithilfe von Fixateursystemen („Kallotasis- Techniken“) bis zum erwartbaren funktionellen Ergebnis aufzuzeigen.

## Introduction

The forearm is a complex anatomical and functional unit with unique osseous, soft tissue, and articular composition. Disruption of this complex biological relation due to posttraumatic changes can have significant impact on the functional system leading to pain, instability in both the proximal and/or distal radioulnar articulation, and reduced range of forearm motion. Corrective osteotomy for malunited fractures or other posttraumatic deformities of the upper extremity, especially in the forearm are challenging procedures and should be performed in specialized centers. In this review we will discuss the essential aspects of anatomy and pathomechanics, clinical and radiological assessment, and the pathway from preoperative planning to the actual deformity correction surgery, either with one-stage correction or using external fixation (“callotasis techniques”) and finally the functional outcome we can expect for our patients.

## Anatomy and pathomechanics of forearm malunion

**Fig. 1:**
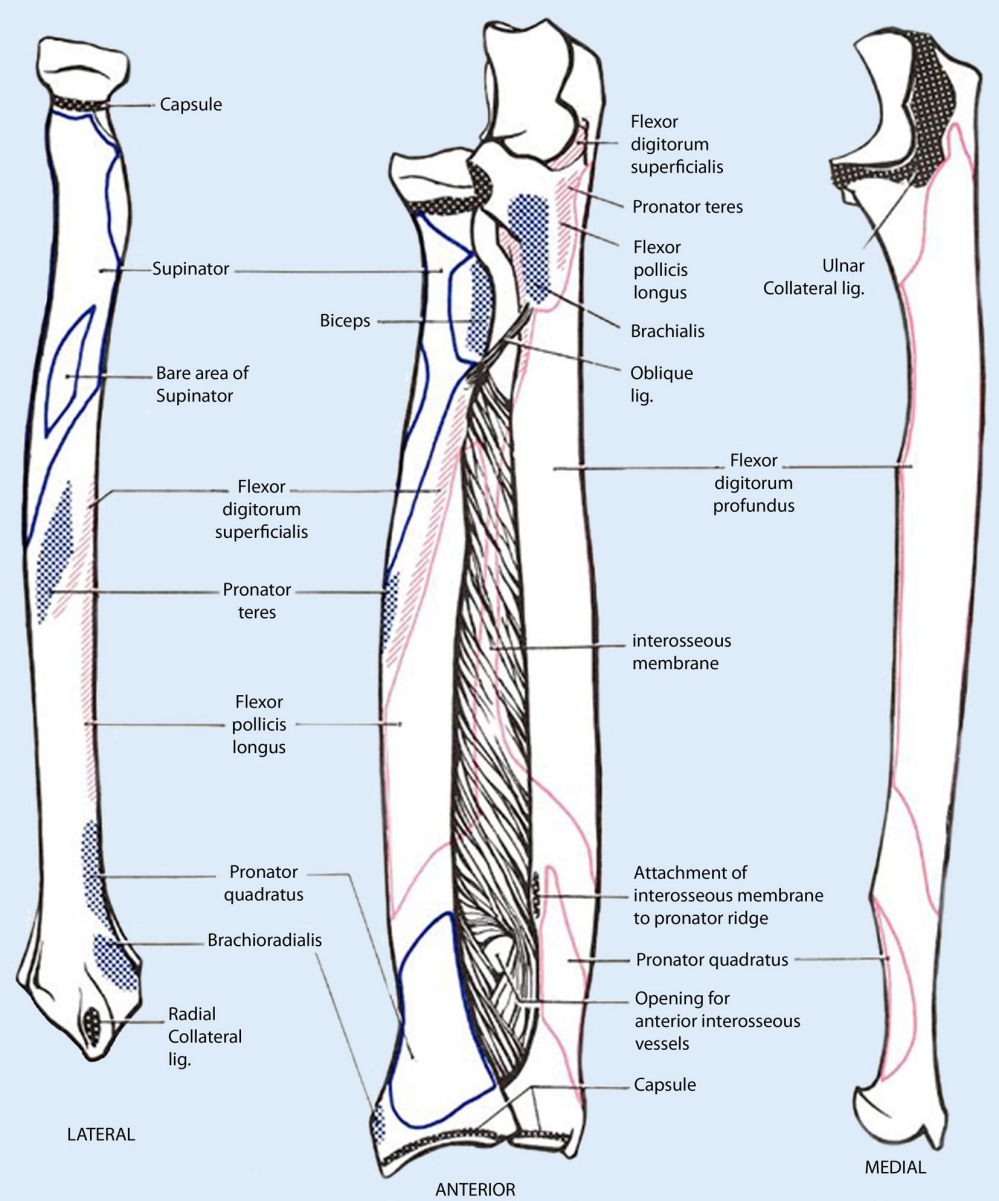
Schematic drawing of both the radius and the ulna (*left* and *right*) and the forearm bones together with the interosseus membrane. The *black* shaded areas represent the capsular insertions, the *blue* and *red* shaded areas show the abundant muscular origins and targeting arease. (Modifyed after DATHMOUTH.edu)

The forearm can be conceptualized as a single bicondylar articulation [13]. In general, the operative treatment of both acute and malunited forearm fractures is in line with the AO principles of restoration of anatomy, stable fracture fixation, and preservation of blood supply with early mobilization. Radius and ulna form a dynamic functional unit with quite astonishing and highly specialised proximal and distal articulations. The radius has a physiological bow (around 7 % and always under 10 % of the total radial length) and rotates around a more or less stationary ulna during pro- and supination [8]. The longitudinal axis of the forearm bisects the center of the radial head and the distal ulnar fovea in the distal radioulnar joint (DRUJ). The interosseous membrane (IOM) between radius and ulna contributes to its longitudinal stability. The central and dorsal oblique bands provide axial and proximal radio-ulnar joint (PRUJ) stability, respectively (Fig. 1). Secondary DRUJ stability is provided by the distal membranous portion [11].

The myriad of different muscles, the supinator, pronator teres and pronator quadratus muscles, the muscles and tendons spanning over the elbow and/or the wrist act as also as (tertiary) stabilizers in this complex construct and act as motors for pain-free delicate motion (Fig. 1). The elaborate course of three main nerves down the forearm and to the wrist hand, especially the radial nerve adds to the challenging anatomy. Normally the PRUJ and DRUJ are the only points of contact between the radius and ulna. The radioulnar articulation is stabilized proximally by the elbow joint capsule and annular ligament and distally by the triangular fibrocartilage complex and its delicate capsule and tendon-sheet network.

The pathomechanics of forearm malunion can be directly related to disruption of the radioulnar relationship, leading to altered motion and potential instability. Morrey et al. showed that performance of most activities of daily life requires 50 ° each of pronation and supination [16]. A higher range of forearm rotation is clearly required and requested for modern activities [8, 14]. The rotational arc is variably impeded by dorsopalmar and radioulnar angular, axial or most often combined deformities [11]. Angular deformities of the radius and ulna increase IOM tension leading to bony impingement and restricted radial rotation about the mechanical axis. Axial rotational deformities also lead to stiffness and restriction of forearm rotation due to malalignment and abnormalities in the radioulnar articulation [2, 18, 22]. Key factors associated with angular deformity include the degree of angulation, location of deformity and the fact if one or both forearm bone involvement [10, 14, 19].

## Clinical presentation and assessment

While posttraumatic forearm malunions may occur after either nonoperative or operative treatment of acute fractures as well as following deformity correction surgery, hereditary conditions are often presenting as visible deformities and impairment of function, often aggravating during growth. Clinical history may elicit pain, stiffness, loss of motion, loss of power and disability as well as visible cosmetic changes. Painful forearm rotation may be related to bony impingement and tensioning of the IOM secondary to angular deformity. It may also occur due to abnormal joint kinematics following radioulnar joint malalignment secondary to axial malunion.

Clinical examination should investigate and objectively record the restriction in range of forearm motion, particularly pronation and supination, and ascertain signs of DRUJ instability and pain with and without movement or strenuous exercise. Painfull clicking at both the elbow and wrist joint during pro- and supination, hard or weak stopping of motion (often with a visible and palpable subluxation of the joint complex, especially at the elbow), and motion and stability of both the wrist and elbow joint will be documented. Already in the early stage information about hobbies, daily restrictions caused by the condition, pain medication use, ability to perform physical exercises and the importance of having a “normal” looking forearm (the cosmetic aspects) should be recorded. The vascular and neurological status, previous scars, a history of previous infection, and skin changes should be documented. In case of neurological impairment, nerve conduction assessment is mandatory. Another important part of the preoperative assessment should focus on information about possible complications, the clinical outcome with conservative (nonoperative) treatment versus an operative intervention and the need of subsequent surgery (possible second stage correction in the same or other region), the expected functional outcome and scope of improvement and finally an evaluation of the compliance of the patient and the family. This also has a profound impact on the treatment concept used; some patients are better served by a one-step correction than a long-lasting gradual correction of a complex deformity with a complex external fixator system.

## Imaging

**Fig. 2:**
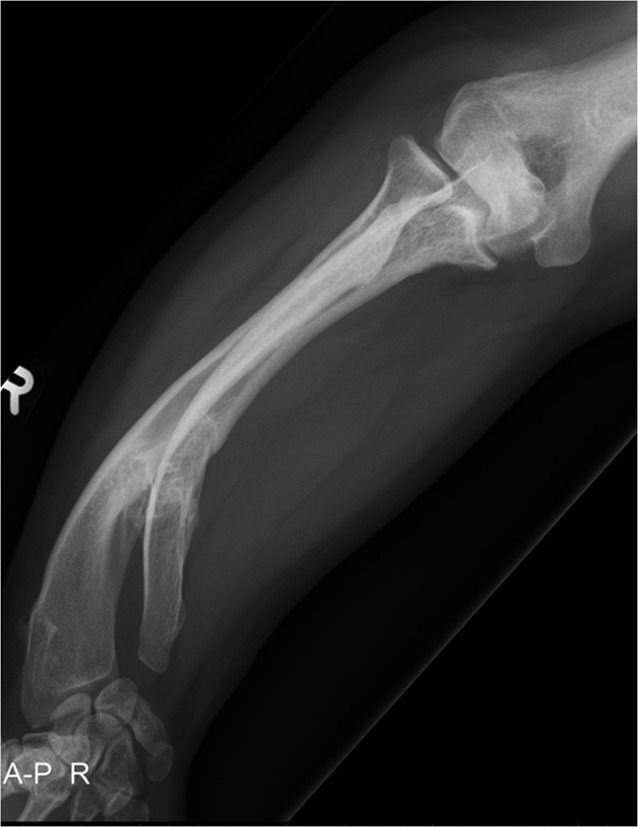
Posteroanterior (PA) radiograph of the forearm in a 15-year-old patient with multiple hereditary osteochondromata (MHO). Although there is a complex three-dimensional deformity in both the distal radius and the ulna with gross visible deformity both the elbow joint and the wrist joint are projected in true PA view

Radiographic assessment includes posteroanterior (PA) and lateral views of the whole forearm (with wrist and elbow) in neutral rotation plus images in maximal pronation and supination. PA radiographs can be obtained with the arm placed on the imaging plate with the shoulder at 90 ° of abduction and elbow at 90° of flexion (if possible). The beam is orthogonally directed toward the forearm in neutral position in the PA direction. Contralateral radiographs should be acquired for preoperative comparison although important side-to-side differences have been demonstrated in healthy populations [24]. Additional wrist and elbow radiographs, centering on the DRUJ and PRUJ in true anteroposterior (AP) and true lateral views are also mandatory. The relative length of the radius and ulna should be measured, including degree of ulnar variance, in comparison with the contralateral side (which in hereditary conditions can be affected too). Complex deformities should be assessed on true AP and lateral views but radiographs are often compromised and good interaction with the radiologic department is mandatory (Fig. 2). Rotational deformities are more difficult if at all to assess and can be assessed using Bindra’s method, that is, the measurement of the relationship between the radial styloid and bicipital tuberosity and ulnar styloid and coronoid process on full forearm views comparing both sides using additional cross-sectional imaging (normally computed tomography (CT) scans, [2]).

**Fig. 3:**
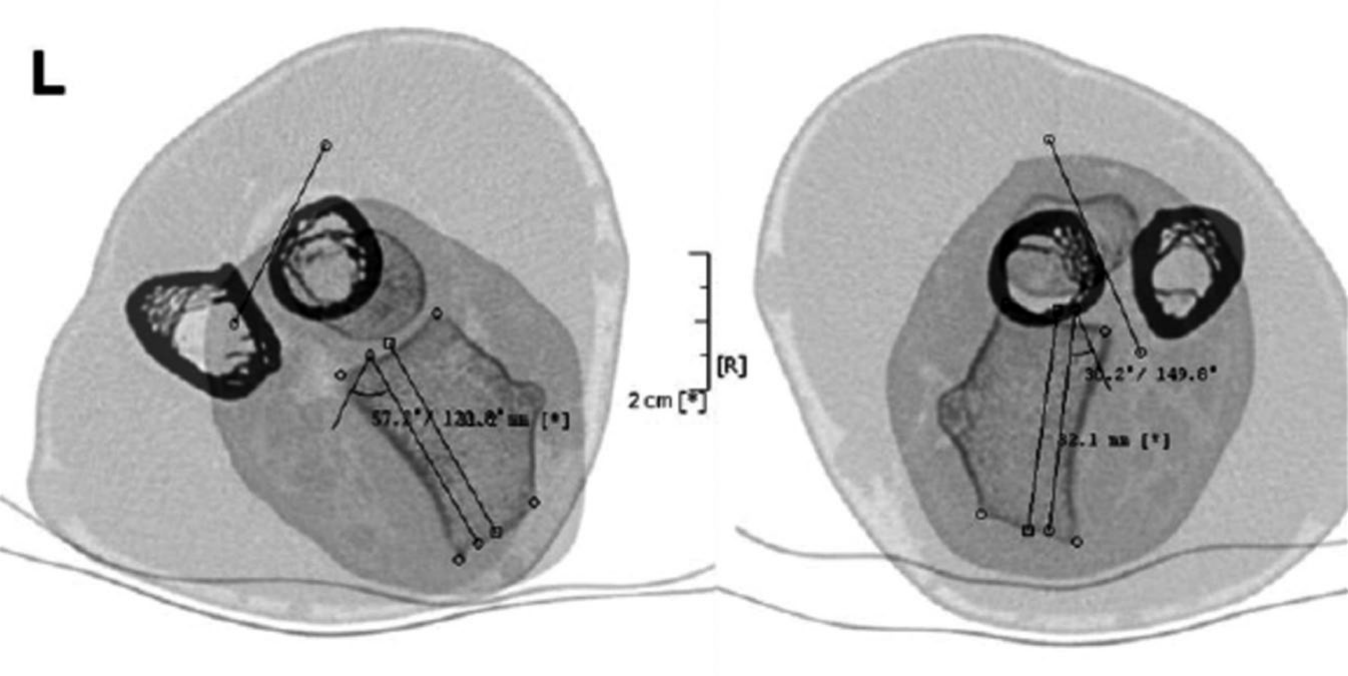
Measurement of the rotational deformity according to the method of Bindra where an increased supination of 27° (*left* side) was found. *Single lines* with *square ends* depict the distal radius and lines with circle ends the proximal radius (bicipital tuberosity); modified after Bindra [4]

**Fig. 4:**
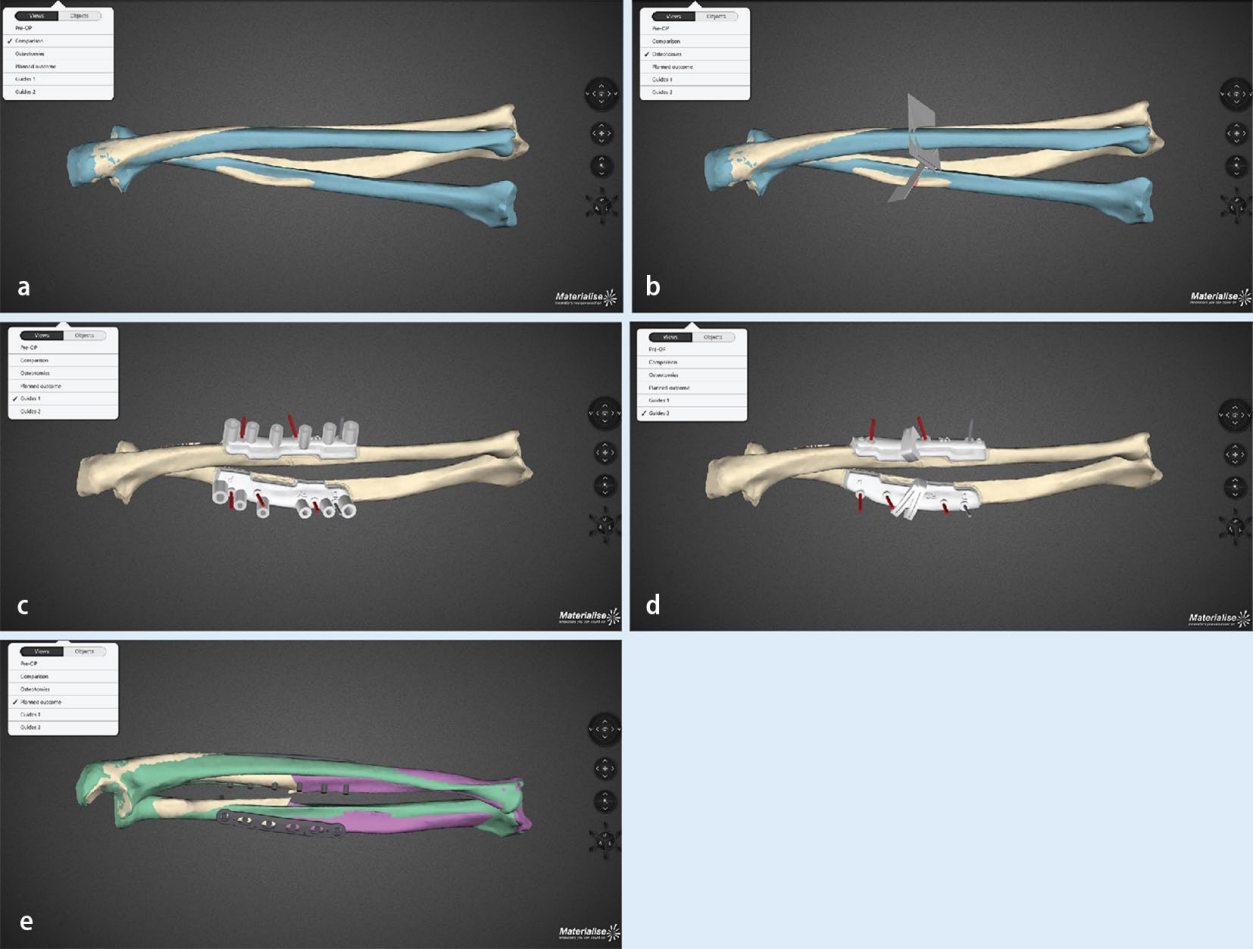
Use of a commercial available software (Materialize®) in a revision case of malunion in the right forearm in a 19-year-old young man, who sustained a forearm fractures as a child: Status after two attempted corrective osteotomies, visible deformity, restricted forearm rotation with 45 ° of pronation and 5 ° of supination. **a** Overlay of both forearms after mirroring of the uninjured left side (*blue*). **b** Placing of osteotomies on the deformed radius and ulna. **c** Simulation of rapid prototyping drill templates on both radius and ulna. **d** Simulation of rapid prototyping osteotomy guides. **e** Simulation of the final surgical result with plates and screws in place, anatomic matching of the deformed side and mirrored uninjured site

Comparative CT may assess rotational deformity more effectively using cross-sectional images in pronation and supination ([2]; Fig. 3). Three-dimensional reformatting allows for visualization of the deformity in a more plastic and “normal” way and is very informative, especially during counseling with the patient and his relatives. Screenshots at different angles can be used to explain the distorted anatomy and after subtracting the radius/ulna or other bones can be effectively used for preoperative planning (Fig. 4). Special scanning protocols of both forearms with the neighboring joints are used for computerized planning templates (see section planning).

## Analyze the deformity and make a planning

**Fig. 5:**
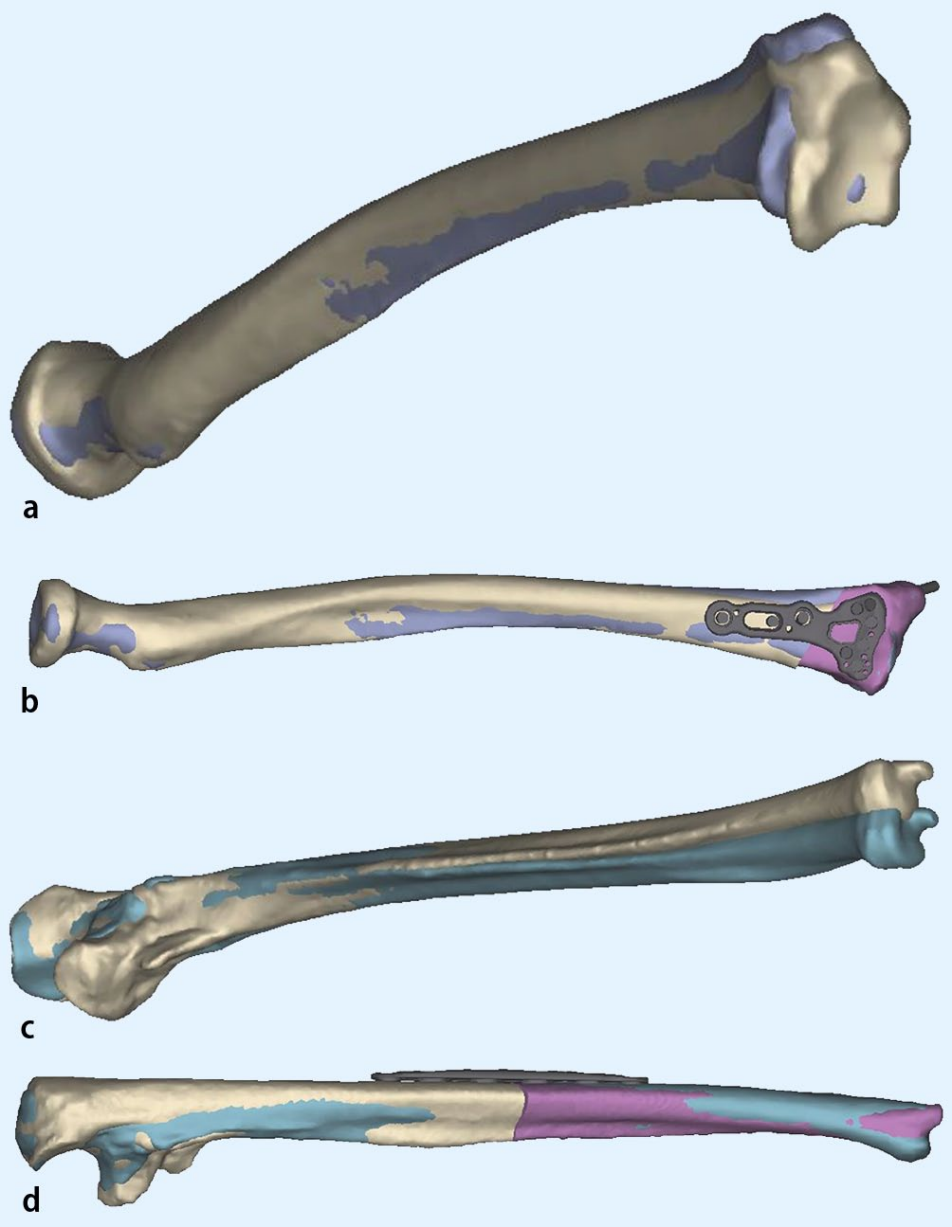
Exemplary printout images from the commercial available software (Materialize®) in a revision case of a 30-year-old female patient with an injury to the left elbow as a child (presumable a radial head dislocation with load transmission over the IOM and a concomitant fracture of the distal forearm, the injury was treated with temporary pinning from the capitulum through the radial head (“Wittsche Spickung”)). Clinically there is painful stop in supination at 0 °. **a** Simulation of the left radius, in *blue* the mirrored healthy *right* side is overprojected demonstrating a shorter and radial translated joint distal radius joint block. **b** Simulation of a radial extraarticular closing wedgen translation osteotomy stabilized with a virutal modern palmar locking plate. **c** Simulation of the left ulna with overlayed mirrored right ulnar showing a ulnarly directed malunion, and (**d**) matching the left ulnar after corrective osteotomy to the normal anatomy of the mirrored right ulna

**Fig. 6:**
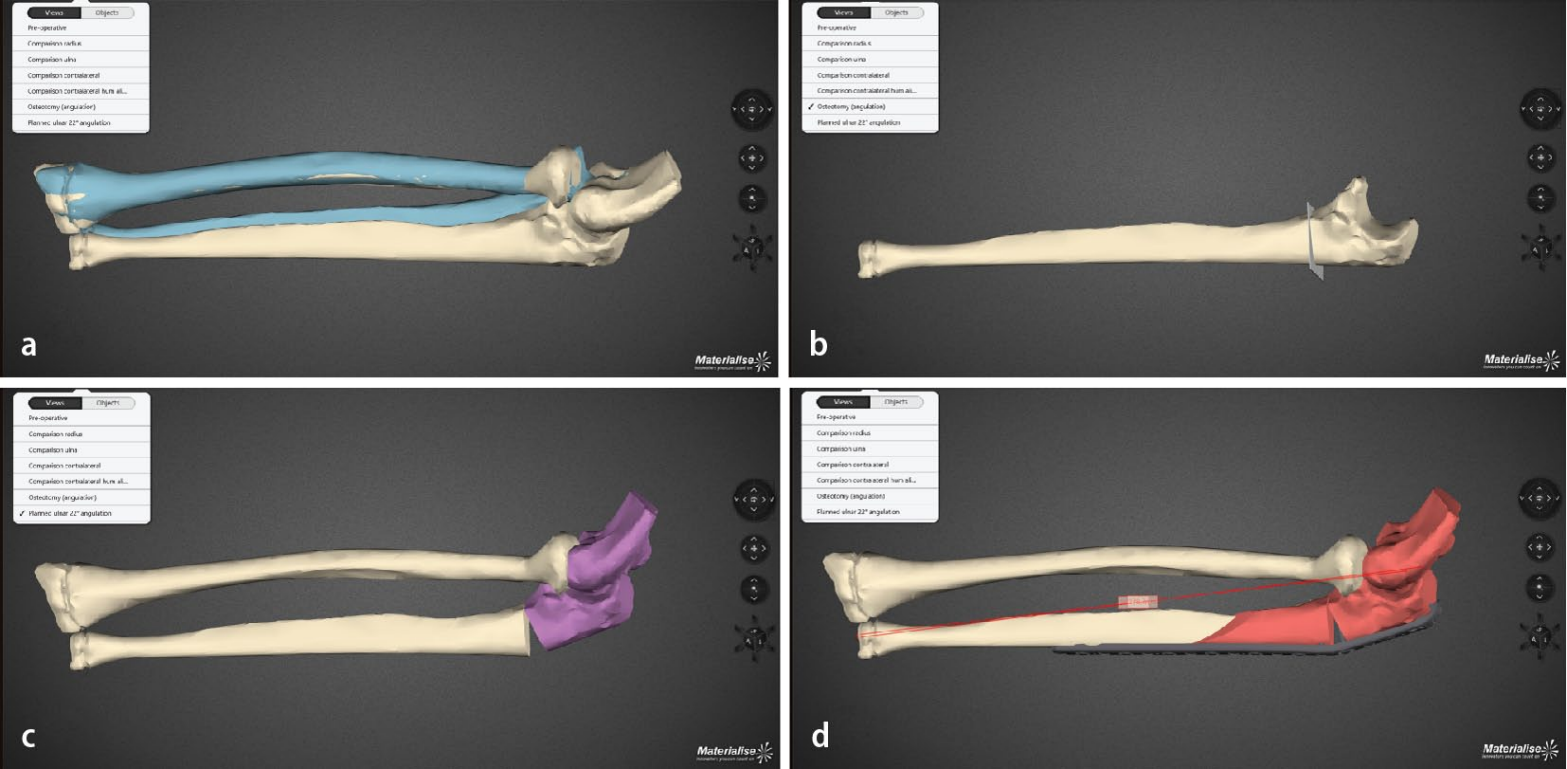
Use of a commercial available software (Materialize®) in a revision case of chronic radial head dislocation in the left elbow in a 15-year-old boy. Complaints are loss of flexion and painful loss of prosupination, previous attempt of reduction of the radial head with a ligament sling without corrective osteotomy (so-called Bell Thawse operation). **a** Preoperative status with anterior superior dislocation of the (already deformed) radial head. In *blue* the mirrored healthy right side is overprojected over the left elbow. **b** Osteotomy plan for the opening wedge osteotomy of the ulna. **c** Simulation of the relocation of the radial head and axis into anatomical position, the radius is still too long and causes impingement on the capitulum. **d** Simulation of an additional sliding oblique osteotomy of the ulna shaft for acute lengthening of the ulna to allow more relative space for the radial head. A modern olecranon plate is virtualized and bent to simulate the intraoperative situs and sound osteosyntesis

Preoperative planning is essential in deformity correction surgery. We strongly recommend to make a printout of the aforementioned forearm radiographs or even better use available scan reconstruction images, do a conception drawing and, following the recommendations of Paley and his group of writing a structured treatment plan with key information on the medical condition/treatment story, patient complaints and functional impairment, the problems (deformity) to address, the planning method used (conventional vs. computerized), the operative protocol (with the different operative steps), the equipment necessary and possible obstacles during or after the surgery [23]. In some countries, for example, in Norway and especially in cases with rotational deformities computerized planning has become a recommended tool and has been employed in these cases by the first author in the last years. It is quite important to state that computerized planning is only an aid and profound knowledge of deformity corrections is an essential prerequisite to treat these patients. Early experience of computer-aided planning has been promising [3, 7, 13, 17, 18, 20, 22, 24]. It has been employed for simulating pre- and postoperative motion and integrated software tools are available now for planning of osteotomies in complex diaphyseal malunions with good functional results using custom-made templates, cutting guides and virtualized implants (which sometimes are also custom made) into the computerized planning ([17, 22, 24]; Fig. 6). For the interested reader we did include the very informative paper by Frame and Huntley on rapid prototyping in orthopaedic surgery [7]. There is one interesting randomized controlled trial in Holland underway comparing computer-assisted with non-computer-assisted (conventional) planning for extra-articular distal radius malunion [13]. Using the DASH- and Patient-Related Wrist Evaluation scores as patient-related outcome measures and grip strength, radiological outcome and patient satisfaction as additional outcome this may shed some light on the usefulness of these technical tool in a clinical setting of posttraumatic pathology with moderate complexity. Our positive experience with using computerized planning comprises a better understanding of the deformity and even in some cases the possibility to offer the patient different treatment modality at a different level of the forearm (Fig. 5).

When analyzing a deformity and even with all the computerized options available we still recommend a basic stepwise approach to analyze the deformity. The concept of overlay drafting using the contralateral forearm as a reference can be used to calculate the degree of correction required if there is no major patholgy in the controlateral side. Nagy et al. utilize these principles in quantifying the orientation of deformity in space by defining the true angle of deformity in angular malalignment [18]. However, malunion with complex three-dimensional deformity of both forearm bones is difficult to assess accurately by means of radiographic or cross-sectional images alone. Especially rotational malalignment is extremitely difficult to detect. Several studies have revealed that two-dimensional planning does not always provide accurate information for complex three-dimensional deformities [17]. We therefore recommend computerized planning in these cases (Figs. 4, 5, 6).

## Operative rationale for posttraumatic deformity correction

The basis of forearm malunion surgery is consistent with the fixation of forearm fractures in general, namely the restoration of length, angular and rotational alignment, and displacement as well as the radial bow. Operative indications include intractable pain, deformity, radioulnar joint instability, functional limitations and restricted forearm range of motion. Forearm rotation is often significantly limited with dorso-palmar or radioulnar angular deformity greater than 15 °, radial malrotation greater than 30 ° and ulnar malrotation greater than 20 ° compared to the contralateral side [8, 10, 19]. The goal of operative intervention is to achieve at least 50 ° of pronation and 50 ° of supination, with meticulous planning and using computerized planning it seems that much better functional results are possible.

## Operative principles

**Fig. 7:**
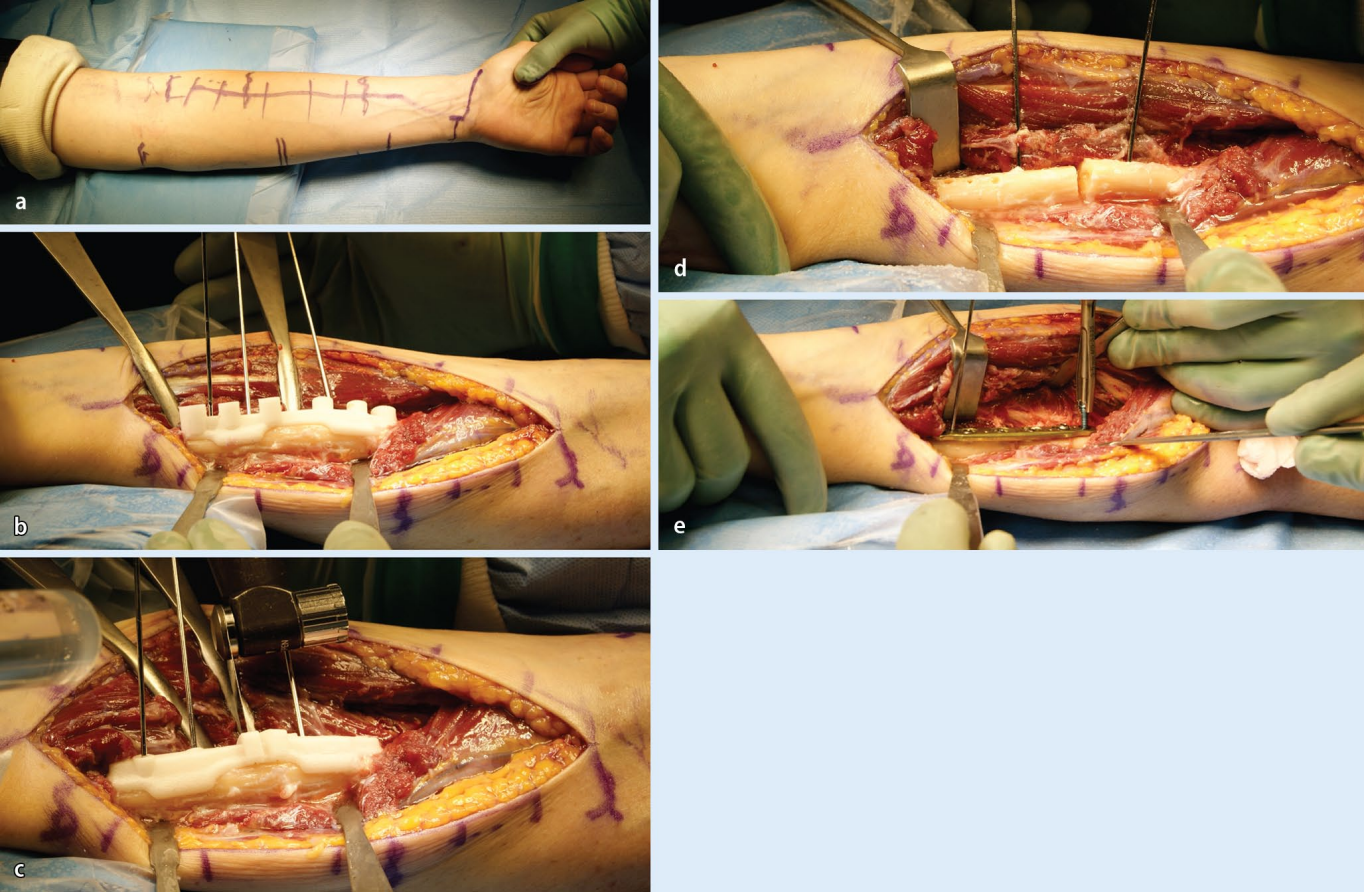
Clinical intraoperative images of the patient, whose planning is depicted in Fig. 4. **a** Marking of the palmar Henry approach to the radius. **b** After visualization of the profound branch of the radial nerve the rapid prototyping drill template is placed on the apex of the deformity. **c** After exchanging of the drill template to the cutting template, the osteotomy is performed. **d** The osteotomy is complete. **e** Sound osteosynthesis with a 6 hole modern osteosynthesis plate, using the predrilled holes to restore anatomy

**Fig. 8:**
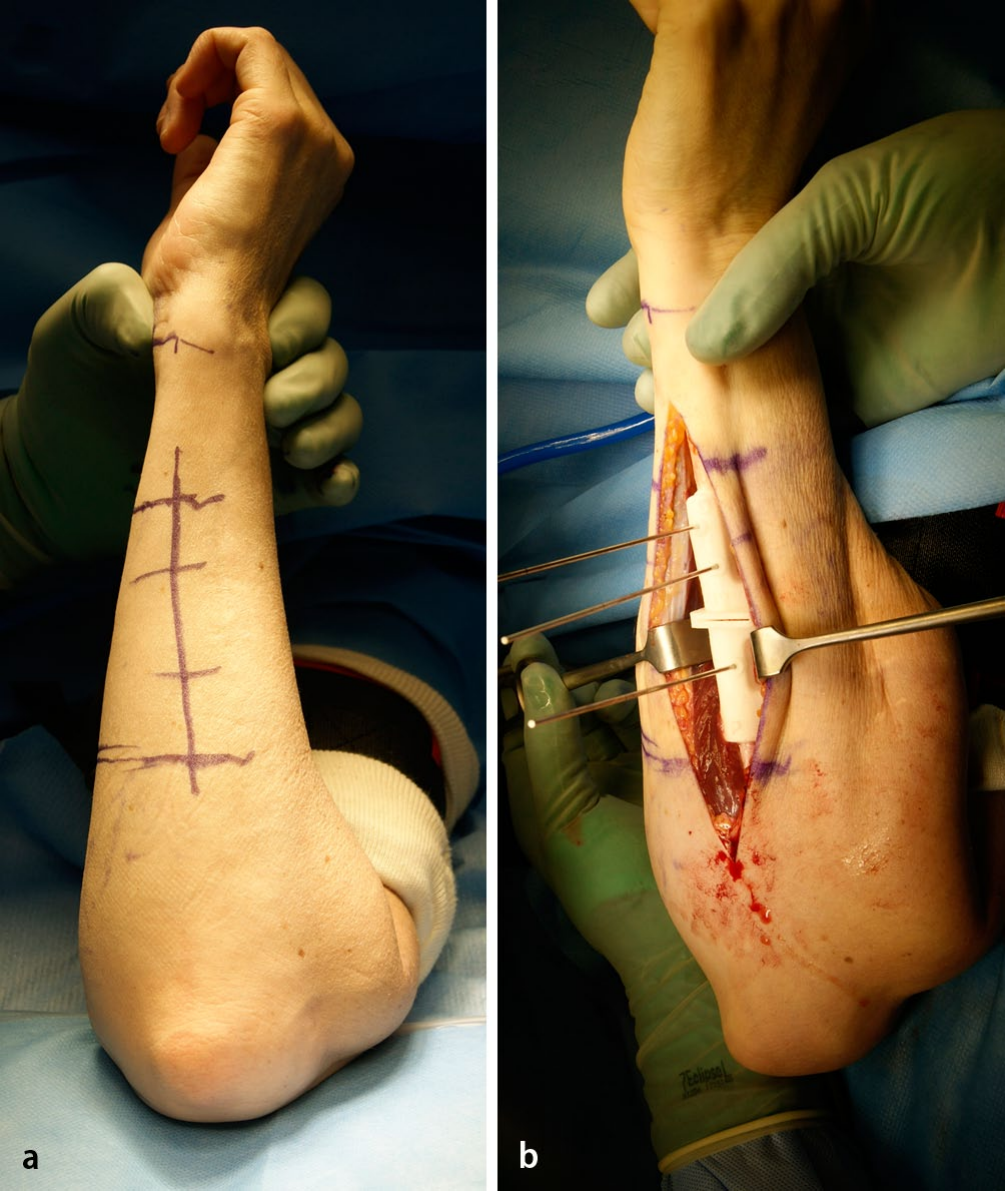
Clinical intraoperative images of the same patient, showing the approach to the ulnar in neutral (standing) position: (**a**) marking of the approach on the skin. **b** The cutting block in situ

Surgical exposure for forearm malunion commonly utilizes the palmar Henry approach and direct ulnar approach (Figs. 7, 8). The radial diaphysis is fully accessed through the release of the pronator teres. Proximal extension allows access to the PRUJ and proximal radius with release of the supinator while obligatory visualizing and protecting the motor branch of the radial nerve with magnifying glasses. The ulna is approached through the interval between the flexor and extensor carpi ulnaris, placing the forearm in neutral position. The approach is fashioned according to the type of osteotomy, which itself is dependent on the type of malunion and correction required, but is generally straight forward. While in the acute trauma setting we recommend to fix the ulna first, in corrective osteotomies of both forearms we start with the radius, using a sterile tourniquet.

**Fig. 9:**
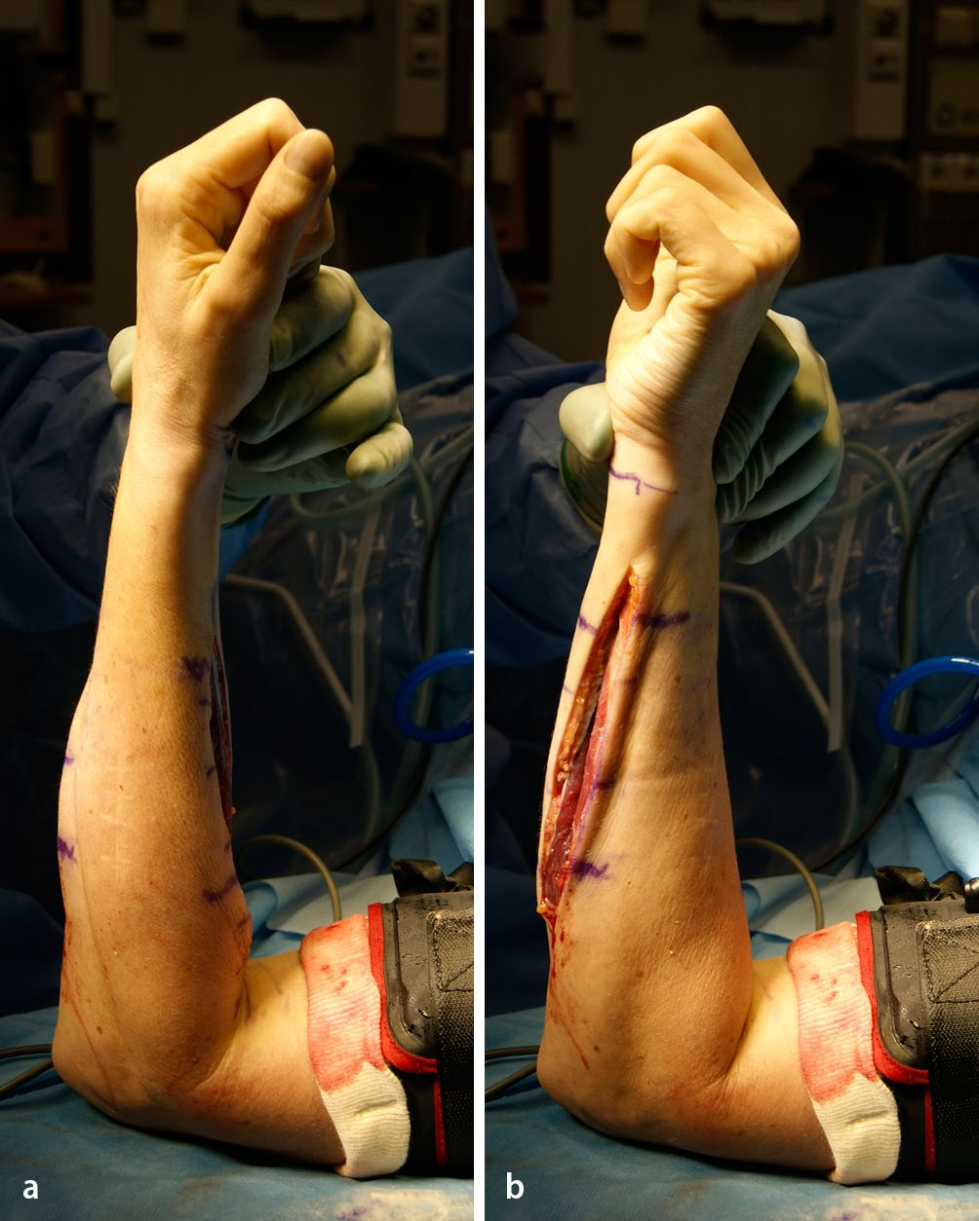
Clinical intraoperative images after correcting the radius and ulna: full passive pro- and supination

**Fig. 10:**
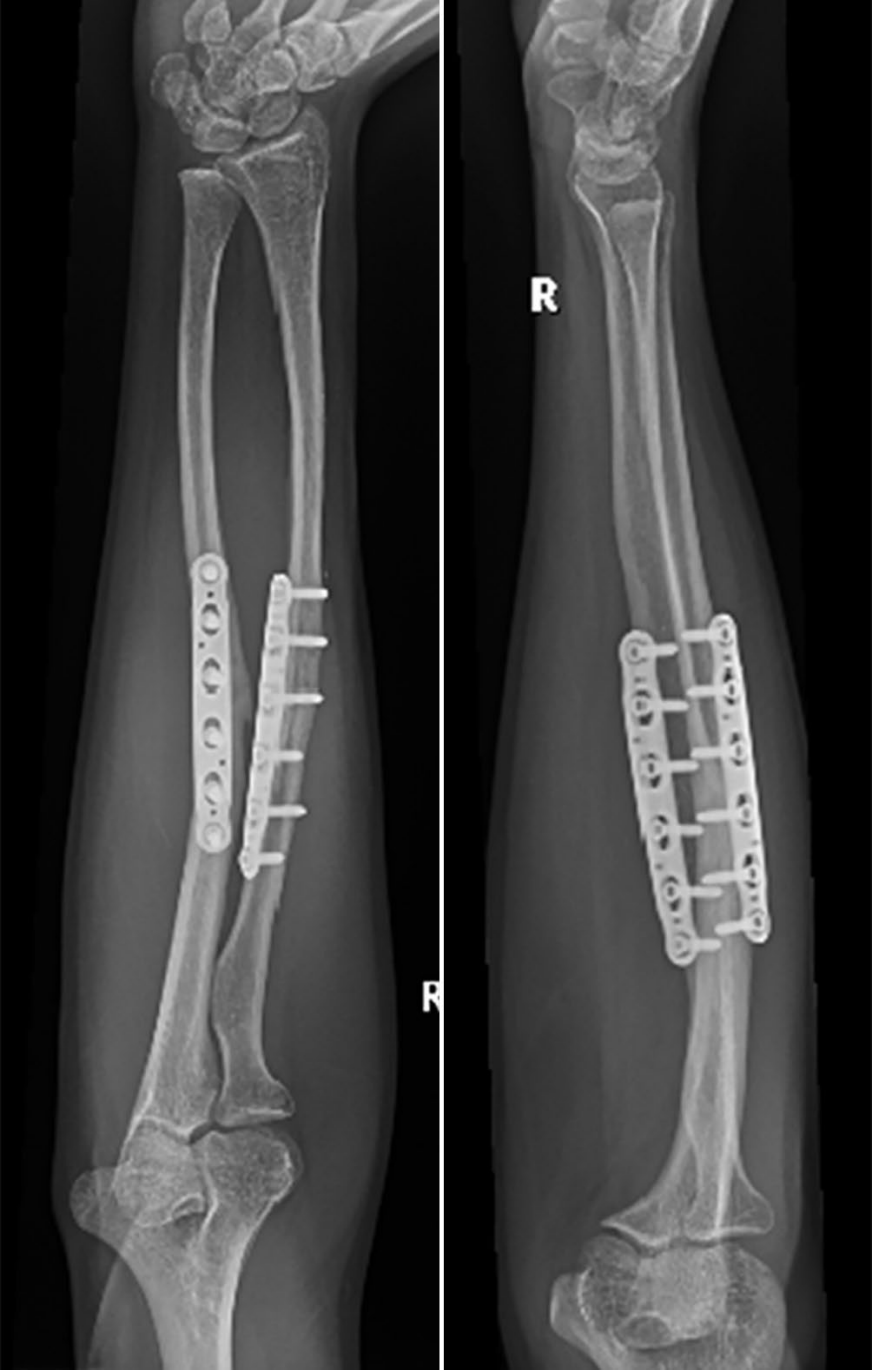
Posteroanterior (PA) and lateral x-rays of the same patient 3 months postoperative demonstrating bone healing of both osteotomies. Clinically is the patient painfree and does have nearly unimpeded prosupination

There are different types of osteotomies, which can be used: transverse (for isolated rotational or translational deformities), oblique osteotomies (for angular corrections with moderate lengthening), “single-cut” osteotomies for complex deformities requiring angular, axial, and length corrections. Residual length discrepancy will require an additional opening or closing wedge osteotomy. When using rapid prototype templates, it is very important to remove all tissue from the area, where to put the template and guard the soft tissue, especially the deep branch of the radial nerve in proximal radius corrections (Fig. 7). At the end of the procedure the passive forearm rotation is documented (Fig. 9). We perform these operations in general anesthesia and perform an immediate check of the soft tissues (possible forearm compartment) and the nerve status in the recovery room. Postoperatively we use a well-padded forearm cast for 2 weeks for pain controll and to keep soft tissues at rest with some support in a cuff and collar sling. Postoperative radiographs x-rays are taken at 1 day, 14 days postoperatively, and at 3 months postoperatively (Fig. 10).

Distraction osteogenesis (“callotasis technique”) is reserved for lengthening of more than 4 cm. We will describe this technique later. In cases with extreme bowing or segmental malunion double-level osteotomies are a good option. The general technique for performing osteotomies involves marking the site of the maximal deformity (CORA or apex), Limited contact compression plates (LC-DCP), 3.5 mm, are contoured and fixed temporarily to the proximal fragment. The plate is then removed, the osteotomy is performed and the wedge excised. Reduction is achieved and checked under fluoroscopy with several views before definitive fixation; sometimes we use fine threaded wires with washers as temporary fixation means. In cases of significant soft tissue contracture sometimes additional external fixator application may also be employed.

## The armentarium

The use of K-wires for rotation control, rapid prototyping templates, modern plates and oscillating saws are just a humble minor part in the armentarium for deformity correction. A huge array of external devices such as monolateral and ring fixator with/ without corrective osteotomy, fixators with swivelling clamps for hemichondrodiatasis procedures, sharp drills for percutaneous or open osteotomies, all packed with their own learning curves and tips and tricks are available. In addition, we can perform a series of salvage procedures, both at the PRUJ and DRUJ; just to name two at the level of the PRUJ, the proximal Sauve Kapandji procedure and the radial head resection and hemiinterposition arthroplasty in cases of arthrosis and chronic radial head dislocation [5, 12]. As stated before, these procedures may be best performed in specialized centers with a dedicated aftercare unit.

Principally we here depict two techniques using clinical examples:

### The use of ring fixators

**Fig. 11:**
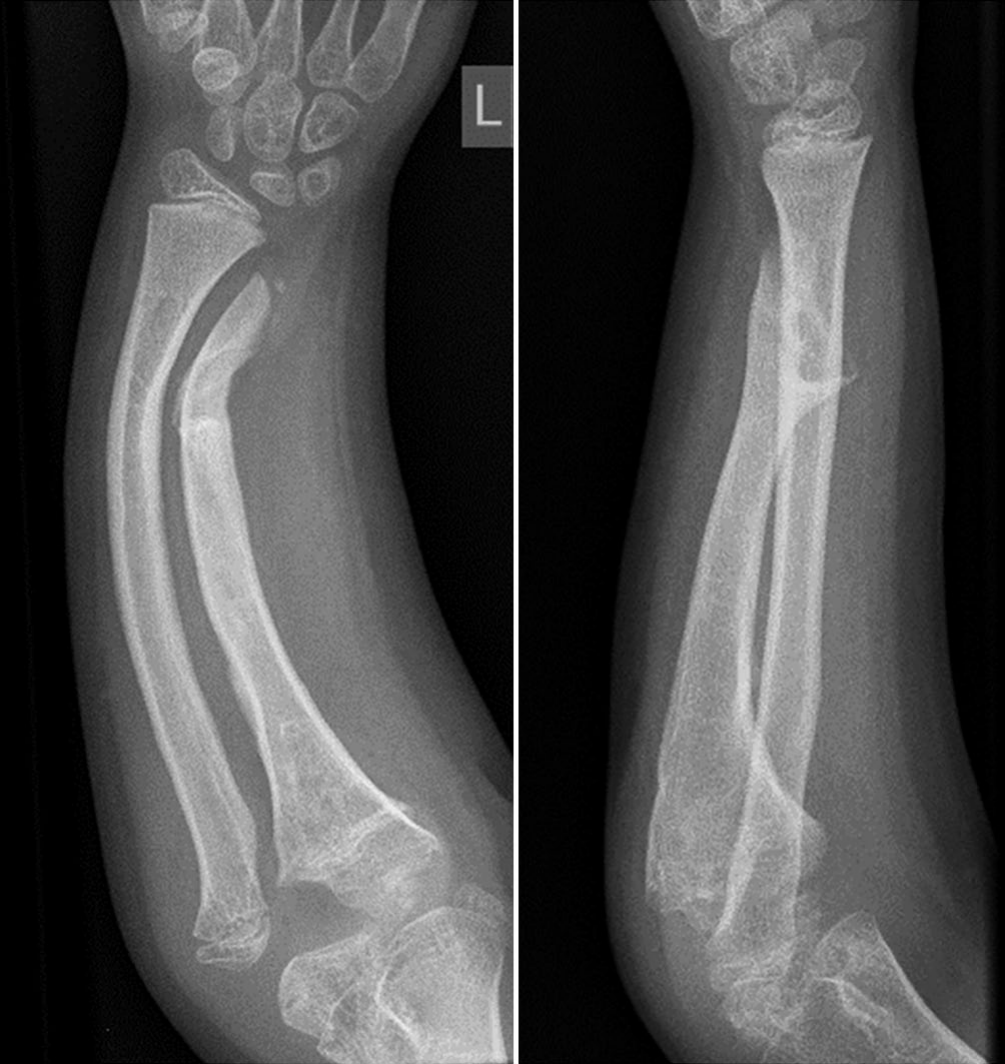
Preoperative x-rays of the left forearm of an 9-year-old with a complex forearm deformity due to focal fibrocartilaginous dysplasia of the ulna leading to secondary deformity of both forearms and consequent dislocation of the radial. The surgical strategy comprises simultaneous correction of the radial and ulna deformities, gradual lengthening of the the ulna and indirect reduction of the radial head at the elbow level using three dedicated rings at the forearm and one additional transmetakarpal ring on the hand. PA and lateral preoperative x-rays; case provided by Durai Nayagam, Royal Liverpool Children’s Hospital, UK

**Fig. 12:**
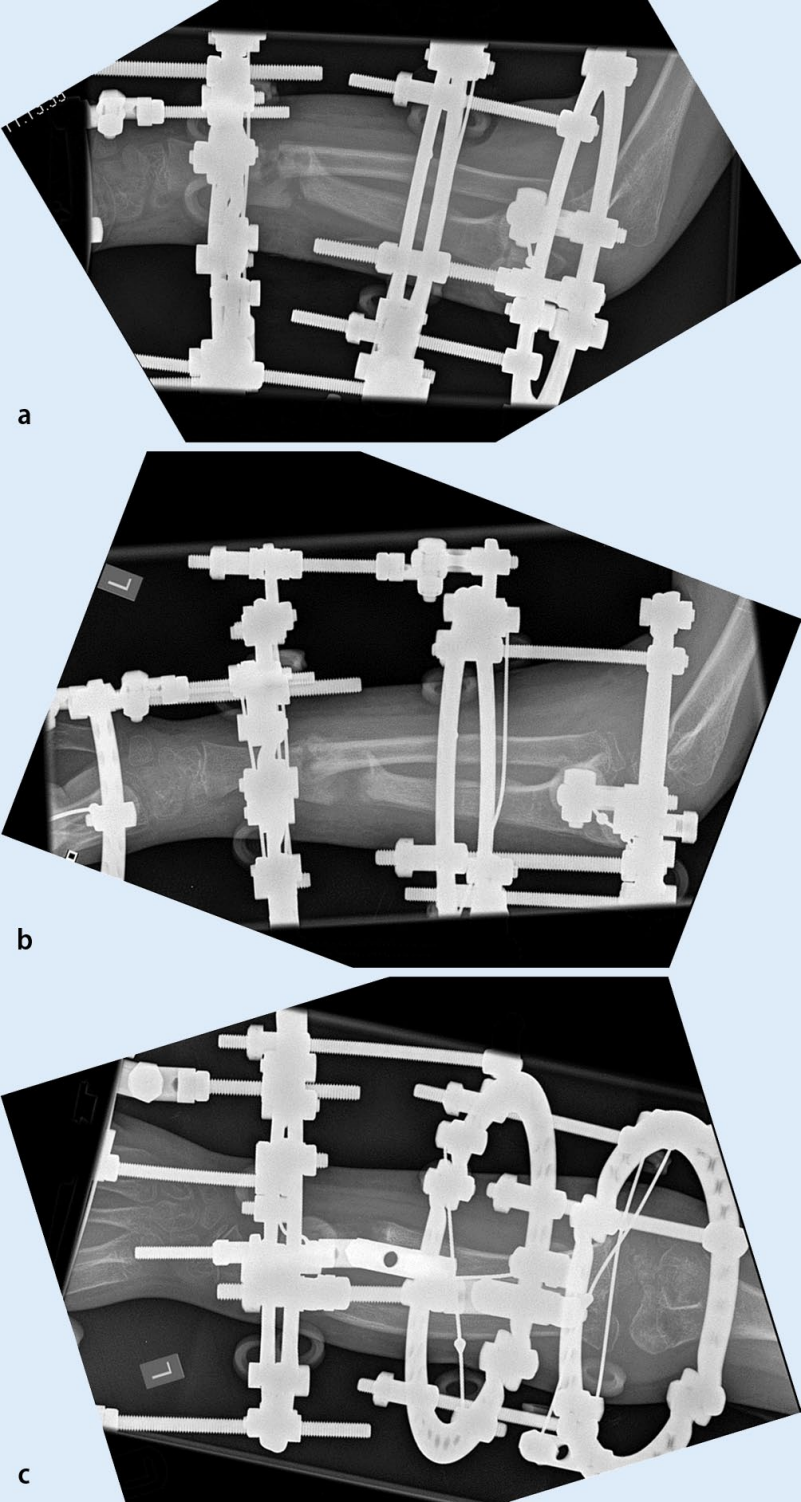
Postoperative x-rays depicting the different corrective steps after mounting of the ring fixator and performing the osteotomies of both forearms. **a** A pure fine-wired Ilizarov fixator is used: in the proximal ring only the ulna is fixated, in the midshaft, only the radius and distal again only the ulna to achieve both correction and relocation of the radial head. **b** Gradual correction of the deformity of the radius and ulna between middle and distal forearm rings. **c** In a second step the ulna is lenghtened by callus distraction between the proximal and middle ring. The radial head reduces when lengthening of the ulna is sufficient

**Fig. 13:**
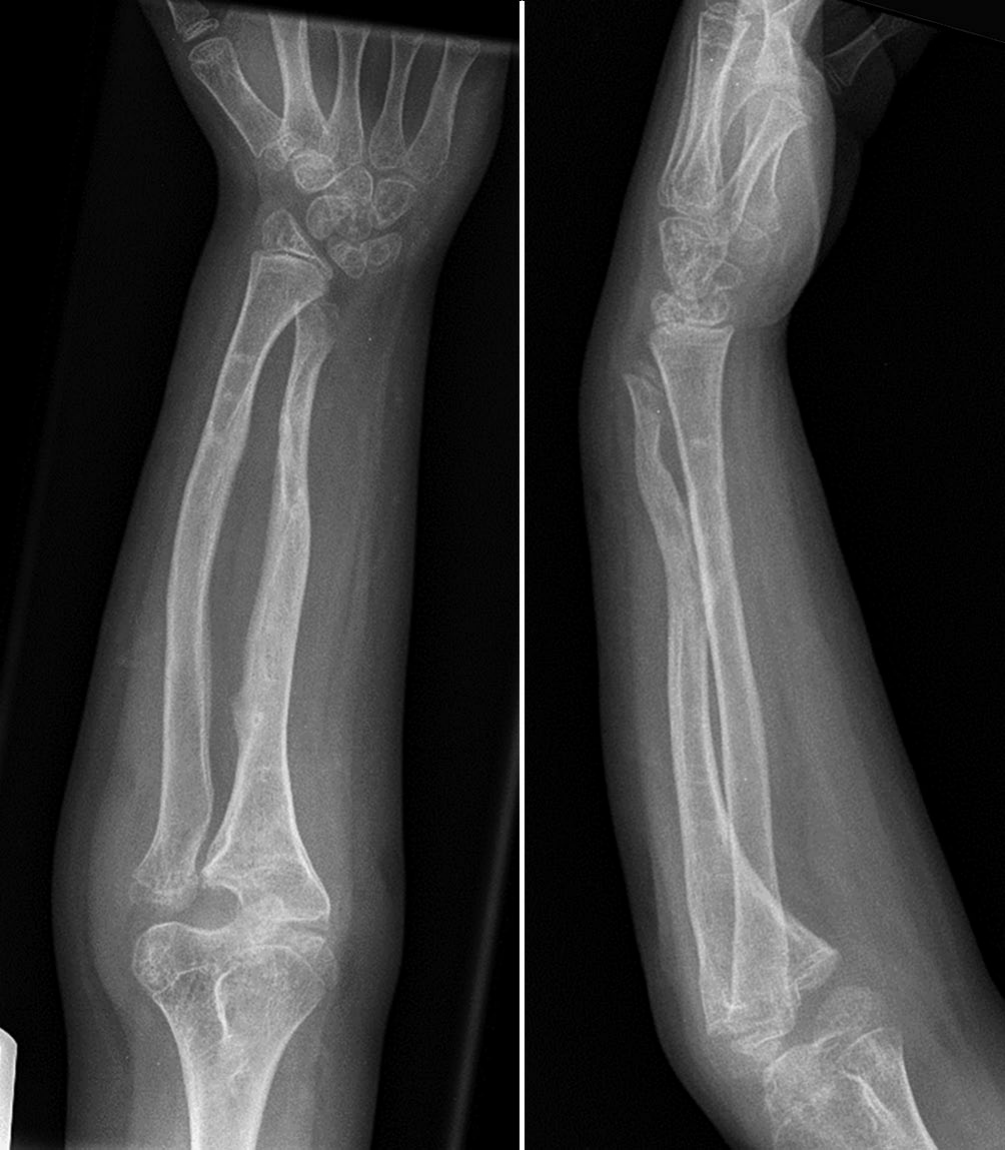
Posteroanterior (PA) and lateral x-rays at intermediate follow-up

**Fig. 14:**
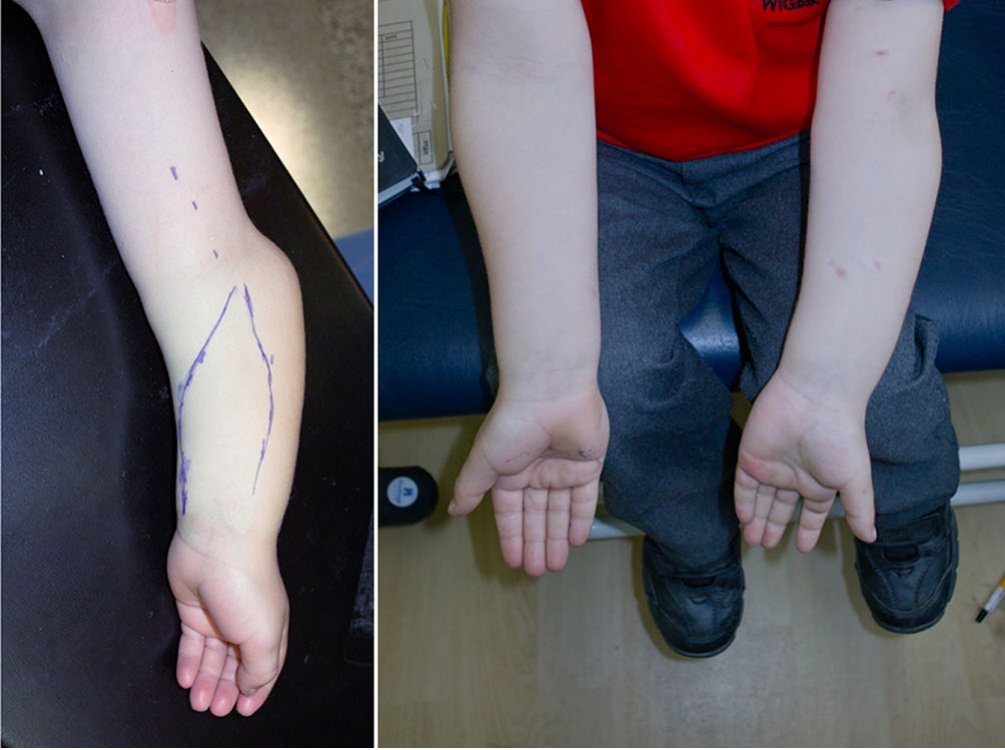
Clinical photograph of the left forearm before the corrective procedure and of both forearms at intermediate follow-up. The young patient was highly satisfyed with both function and cosmetic results

Circular external fixation, and still the Ilizarov ring fixator being the gold standard, in pediatrics has advantages and disadvantages [21, 23]. The advantages include: axial loading of the long bone segment to allow for better bone formation and consolidation by tensioned wires (especially in the non-load bearing upper extremity), fixation of small and/osteopenic bone fragments, a very high degree of adaptability, and the possibility to extend the apparatus across joints (elbow and wrist in our setup) to address instability or subluxation during lengthening [23]. Next to the classic Ilizarov frame, newer frames with software-guided assistant systems such as the Taylor Spacial Frame and the Hexapod have become available [5]. The use of ring fixators is difficult and belongs really into the hands of the superspecialist as it needs a highly trained knowledge of the device, its possibilities and danger, a profound understanding of the cross-sectional anatomy of the forearm and a dedicated aftercare unit. There is a clear interference with activities of daily living, the child has to wear an intimidating device for quite a long time, to pay a lot of outpatient visits and the sometimes painfull distractions can lead to profound psychosocial issues [21]. Bearing these factors in mind, ring fixators are an astonishing treatment module for the very complex and challening corrections, both in posttraumatic and hereditary deformities ([5, 9, 21, 23]; Figs. 11, 12, 13, 14).

#### Hemichondrodiastasis

**Fig. 15:**
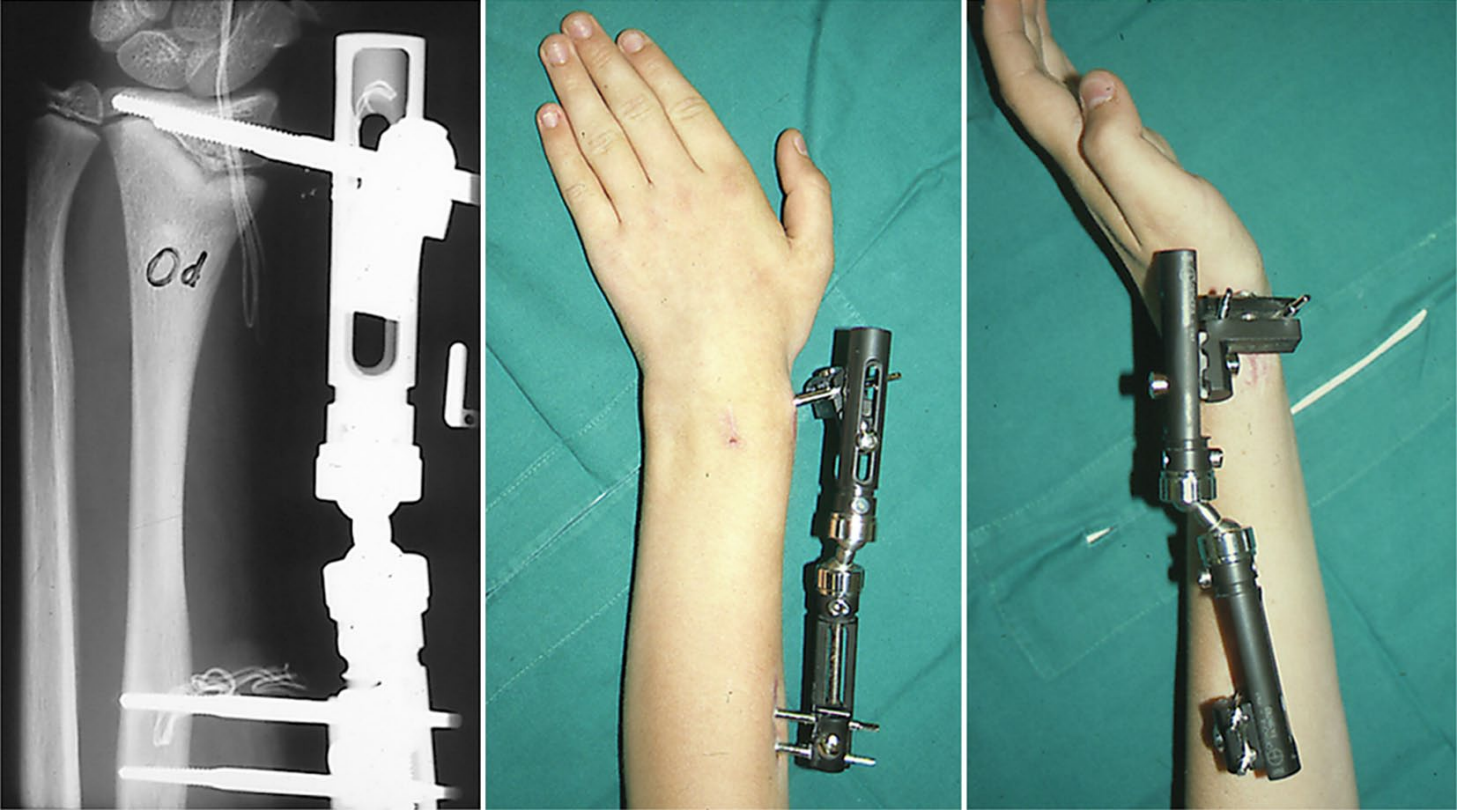
Postoperative x-ray in a patient with posttraumatic limited closure of the central radial part of the growth plate of the left radius in a 15-year-old boy after previous forearm fracture and osteosynthesis using Rush rods. An extraarticular monolateral fixator is applyed with pins in the radius epiphysis distal to the growth plate and into the radius shaft. The clinical pictures on the right do show the position of the fixator after the hemichondrodiatasis procedure. Distraction was performed using the inbuilt distractor in the proximal part of the fixator and by leaving the central distal screw open the fixator clamp distally has rotated by a swiveling mechanism

This is a special technique of closed, gradual, asymmetric distraction of the growth plate to correct angular deformities in growing children using monolateral external fixation with a special swiveling clamp. It was described by Roberto Aldegheri et al. in 1989 in the lower extremity, but can also be adapted to posttraumatic deformities in the distal radius with partial growth plate fusion [1]. The best results are achieved in posttraumatic deformities when the bone bridge occupies less than 20 –30 % of the epiphyseal growth plate. The procedure is best performed toward the end of growth, or earlier if a progressive deformity exceeds 15–20 °. We use a monolateral external fixator with a swiveling clamp at the level of the growth plate at the distal radius (Fig. 15 **and** 16) and combine the distraction of the growth plate with a localized chiseling of the bone bridge using a limited surgical approach.

**Fig. 16:**
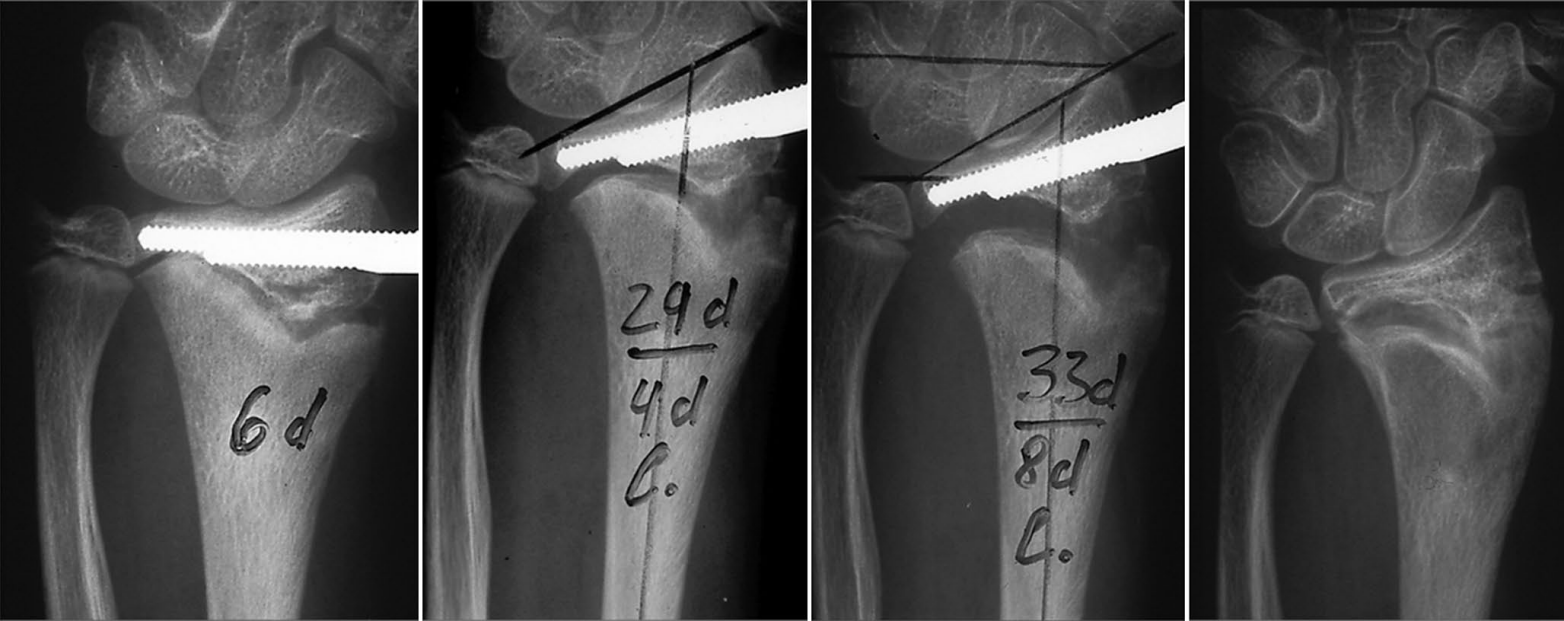
Sequence of postoperative posteroanterior (PA) x-rays showing gradual correction of the radial articular angle and radius length by hemichondrodiatasis after chiseling of the scar in the growth plate of the distal radius

## Functional outcome and conclusion

A simple malunion of the distal radius is associated with a clearly detectable arm-related disability regardless of age [3]. If even in a not complex corrective situation we can document a negative effect of malunion on patient-related outcome we can accept a substantial benefit for the functional outcome in more complex deformities, if we do not place additional burden to our patients by possible complications. Using the modern armentarium described, operating the right patients with the right indication gives very gratifying results. Recent publications show clearly that in children and adolescents (and also in adults) malunited fractures of the forearm can be adequately treated by osteotomy and plate fixation with excellent functional results with minimal complications in an early elective setting [4, 8, 10, 15, 18, 22]. The new computer-assisted techniques, when used cautiously, might even add to the this positive functional outcome [13].
